# Editorial Notes

**Published:** 1917-07

**Authors:** 


### Editorial Notes.

HEARTY congratulations to **Captain Alfred G. T. Fisher,** R.A.M.C. (T.) on his winning the Military Cross for services in the field. Captain Fisher is the second son of Canon Fisher, Vicar of St. Francis, Ashton Gate, and is Demonstrator of Anatomy in the University of Bristol.

**Major W. C. Swayne,** who contributes an original article to this issue of the *Journal,* deservedly received a warm welcome on his resuming his work as Professor of Obstetric Medicine at the University and Obstetric Physician at the Bristol Royal Infirmary. Though he has been hard at work again in our midst for over six months, his good services in connection with the R.F.A. battery, and later as O.C. of a New Zealand battery on Salisbury Plain, will always be remembered.

**Food at School Ages.**

THE Medical Officer of Health for Bristol, Lieut.-Col. Davies, sends us the following notes, which it will be felt are worthy of careful note. Recent experience in revising the dietary of an Institution for growing boys—from 12 to 16—has warned me that considerable, perhaps lifelong, injury might be caused to growing boys and girls at these ages by misapplied enthusiasm in the direction of food saving. This period of life is, as Burney Yeo points out, "one of the most critical and important epochs in the life of the individual as regards sufficient and adequate nutrition ; it is a time of active growth and development, both physical and intellectual, and it is a time when any serious check to the perfect and complete evolution of the organs and functions of the body may lead to ineradicable mischief, and severely handicap the individual in the subsequent ' struggle for existence.' " Especially, one may add, as the prospective parents of the race.

Pamphlet No. 7, published by the Ministry of Food, points out: " Children need plentiful food for three reasons; their surface is large compared to their weight, they are growing, and they are generally very active. A child of eight needs half as much as a grown-up, a child of twelve three-fifths as much. A girl of sixteen needs as much as her mother, and a boy of sixteen may eat as much as his father."

The number of calories (heat units) that should be supplied daily by the food taken is laid down thus (Pamphlet No. 7) :—

| | *Calories.* |
|---|---|
| " Men, sedentary, and boys over 16 | 2,500—2,800 |
| Women, in business, and girls over 13 .. .. .. .. .. | 2,000—2,200 |

A tall, thin person will need 200 or 300 calories more than a plump person."

If children of school ages are rationed, Managers and Medical Officers should see that the necessary number of heat units are supplied by the food. The Ministry of Food Pamphlet No. 7 (*National Rations and how to supplement them,* by Dr. Spriggs) gives a table which renders the calculation of dietary values absolutely simple.

The Ministry of Food never intended to stint the food of growing adolescents. Elder children are an exception to the four-pound ration. In a written reply to Mr. Duncan Millar, Captain Bathurst supplies the following table of what is considered sufficient weekly ration of bread, meat, and sugar for growing children :—

| | *Bread.* | *Meat.* | *Sugar.* |
|---|---|---|---|
| Under 10 .. .. .. | 3 lbs. | $1\frac{1}{4}$ lbs. | $\frac{1}{2}$ lb. |
| 10—12 .. .. .. | 4 lbs. | 2 lbs. | $\frac{1}{2}$ lb. |
| 13—18 .. .. .. | 5 lbs. | $2\frac{1}{2}$ lbs. | $\frac{1}{2}$ lb. |

Finally, to quote the Ministry of Food Pamphlet No. 21 : "Everyone will agree that the greatest care must be taken to give our children the food they need, whoever else goes short. Upon them will lie the duty of holding what has been won by the blood of the brave. They must be fit in body and mind to make peace more glorious than war by the right use of our dear-bought freedom."

**Lieut.-Col. Prowse, O.C. 2nd Southern General Hospital.**

In the absence on duty in France of Colonel Bush, C.M.G., the command of the 2nd Southern General Hospital has been conferred on Lieut.-Col. Prowse, who is Senior Physician to the Bristol Royal Infirmary. Long before the war broke out he had been recognised as an able administrator by those who knew him in private life, and Lieut.-Col. Prowse's exceptional capabilities in this direction had already proved so invaluable at the 2nd Southern Hospital that when some few months ago it became necessary to find a new administrator, there was little room for doubt as to who was the man for this responsible work. Now we warmly congratulate him on receiving his Brevet-Colonelcy, and we offer our best wishes to the O.C.

**Lieut.-Col. Bush, C.M.G.,** now in France, has been gazetted Brevet-Colonel in recognition of his work at the Front.

**Capt. J. A. Nixon, R.A.M.C.(T.),** must be congratulated on being mentioned in dispatches.

